# A novel approach to explore Safety-I and Safety-II perspectives in in situ simulations—the structured what if functional resonance analysis methodology

**DOI:** 10.1186/s41077-021-00166-0

**Published:** 2021-06-05

**Authors:** Ralph James MacKinnon, Karin Pukk-Härenstam, Christopher Kennedy, Erik Hollnagel, David Slater

**Affiliations:** 1 Department of Learning, Informatics, Management and Ethics, Karolinska Institutet, Tomtebodavägen 18a, 171 65 Solna, Sweden; 2 Department of Paediatric Anaesthesia, Royal Manchester Children’s Hospital, Manchester, UK; 3 Paediatric Emergency Department, Karolinska University Hospital, Solna, Sweden; 4 Paediatric Emergency Department, Children’s Mercy Hospital Kansas City, Kansas City, USA; 5 Department of Patient Safety, Jönköping Academy, School of Health and Welfare, Jönköping University, SE-551 11 Jönköping, Sweden; 6 Institute of Energy, School of Engineering, Cardiff University, Cardiff, UK

**Keywords:** Simulation, Patient safety, Trauma, Training, Emergency medicine, Paediatrics

## Abstract

**Objectives:**

With ever increasingly complex healthcare settings, technology enhanced simulation (TES) is well positioned to explore all perspectives to enhance patient safety and patient outcomes. Analysis from a Safety-II stance requires identification of human adjustments in daily work that are key to maintaining safety. The aim of this paper is to describe an approach to explore the consequences of human variability from a Safety-II perspective and describe the added value of this to TES.

**Methods:**

The reader is guided through a novel application of functional resonance analysis methodology (FRAM), a method to analyse how a system or activity is affected by human variability, to explore human adaptations observed in in situ simulations (ISS). The structured applicability of this novel approach to TES is described by application to empirical data from the standardised ISS management of paediatric time critical head injuries (TCHI).

**Results:**

A case series is presented to illustrate the step-wise observation of key timings during ISSs, the construction of FRAM models and the visualisation of the propagation of human adaptations through the FRAM models. The key functions/actions that ensure the propagation are visible, as are the sequelae of the adaptations.

**Conclusions:**

The approach as described in this paper is a first step to illuminating how to explore, analyse and observe the consequences of positive and negative human adaptations within simulated complex systems. This provides TES with a structured methodology to visualise and reflect upon both Safety-I and Safety-II perspectives to enhance patient safety and patient outcomes.

**Supplementary Information:**

The online version contains supplementary material available at 10.1186/s41077-021-00166-0.

## Introduction

For over 20 years now, technology enhanced simulation (TES) has been a lightning-rod to promote reflection [1], and discussions of how to improve care, aiming to both enhance patient safety and patient outcomes, within increasingly complex healthcare settings [2, 3]. Up to the last decade, the standard response to managing this has been to try to eliminate the complexity and reduce human variability through standardisation of procedures protocolisation and training uniformity [4]. More recent approaches have focused upon embracing the complexity and developing tools to cope and manage it successfully [5]. As complexity in healthcare has increased, so have concepts of safety management and an understanding of the impact of human variability on this. The prevailing starting point that patient safety was considered as an absence of incidents, accidents or a state with the minimal acceptable level of risk, has led to concepts of Safety-I and Safety-II [6]. Safety-I has been defined as a state where “as few things as possible go wrong” in a system. The Safety-II perspective focuses on ensuring “ as many things as possible go well” in the system [6]. The divergence of these two perceptions is arguably the core focus of attention of TES practitioners aiming to enhance patient safety. That is how humans and their variable actions amidst a complex socio-technical system are observed, how their actions are considered, understood and acted upon. Central to Safety-II thinking is that humans and their adjustments, as they work, are vital to maintain safety [6]. Translated to healthcare, this means that things do not go well because individuals behave exactly as they are supposed to, in terms of following protocols, but that safety and positive outcomes are achieved by individuals adjusting their actions and adapting to match the complex scenarios in which they find themselves [6]. As the complexity increases, the human adaptions become more important for system resilience to maintain the desired outcomes. The Safety-II perspective is that of developing an “understanding how things usually go right, since this is the basis for explaining how things occasionally go wrong” [6]. This approach poses significant challenges for TES.

Historically, TES and debriefing strategies have transitioned from exploring individual knowledge, skill or behaviour deficits, to embrace complexity and provide opportunities for improvement with a systems focused approach [7, 8]. Conceptual frameworks, including the Systems Engineering Initiative for Patient Safety 2.0 model [9], that explore work systems, processes and outcomes have shaped system focused simulations to explore gaps in different components of healthcare systems [7]. One example of this is in situ simulations (ISS) that alert teams in the workplace to respond as per their normal practice to simulations, using real equipment and fully implementing care processes [10–12]. These system focused simulations, combined with system focused debriefing frameworks, identify gaps in the healthcare systems that predispose to medical errors [8]. TES has been successful with its clear goals to identify errors, or safety threats within the system explored, that threaten the quality of care and patient safety [7, 12, 13].

The TES approach to identifying gaps, or deficits, that in turn stimulate the implementation of mitigation strategies, and the improvement of patient safety, can be interpreted as ensuring a state of safety where “as few things as possible go wrong”. From this perspective TES as a pedagogy, can be viewed as aligned to aspects of Safety-I thinking [6]; however, debriefing strategies in TES traditionally promote reflection on what went well in addition to what went wrong [14]. If one accepts there is value in exploring both Safety-I & Safety-II concepts with TES, the question then arises of how to utilise TES to understand more about how things go right, in addition to how things go wrong?

In addition to identifying gaps, TES groups have also approached the challenges of understanding and promoting deeper reflection on things that go right [15]. One TES example, the Learning From Success (LFS) approach, provides a framework to explore how teams adapted, what triggered adaptations and why the adaptations made sense [15]. Such a combined approach with TES hinges on analysing human performance adjustments and performance variability, as from a Safety-II perspective, “these are considered normal, necessary and the reason for both acceptable and unacceptable outcomes” [6]. From a Safety-II perspective, a distinction is made between work-as-imagined (WAI), a task is performed matching a pre-set design or protocol, and work-as-done (WAD) how the task is actually managed in the workplace [16]. The Safety-II approach emphasises that the system is made reliable and safety is enhanced by the everyday normal human performance adjustments and performance variability that forms WAD [17]. The LFS approach emphasises the need to simulate to capture and reflect upon these normal “mundane” adaptations of WAD [15]. With LFS, there is the focus on when things go right, not necessarily wrong, as the normal variation of adaptations to unfolding events in simulations are based upon the previous experiences of those participating in the simulations to ensure things go right [15]. Then, by reflecting on these normal adaptations, deeper insights on the rationale behind the normal practice, which is often learnt without reflecting upon it, can be triggered [15]. The challenge for TES to embrace Safety-II perspectives, is to actually see and analyse normal mundane human adaptations that improve care on a daily basis, as they do not provoke the same level of cognition and mental processing as when things go wrong [15]. Without an understanding of why these normal adaptations occurred and importantly the downstream consequences, or repercussions of the actions, it is difficult to reflect upon and relate them to real-life situations [4, 15].

There is a need for a methodology that supports TES practitioners, safety scientists and human factors practitioners to bridge the current gap of how to see and analyse events systematically, when events go right and when they go wrong, taking into account the complexities of the real world. This would then afford the opportunity to embed variations in human practice into future real-life designs, should the adaptations prove beneficial.

**Fig. 1:**
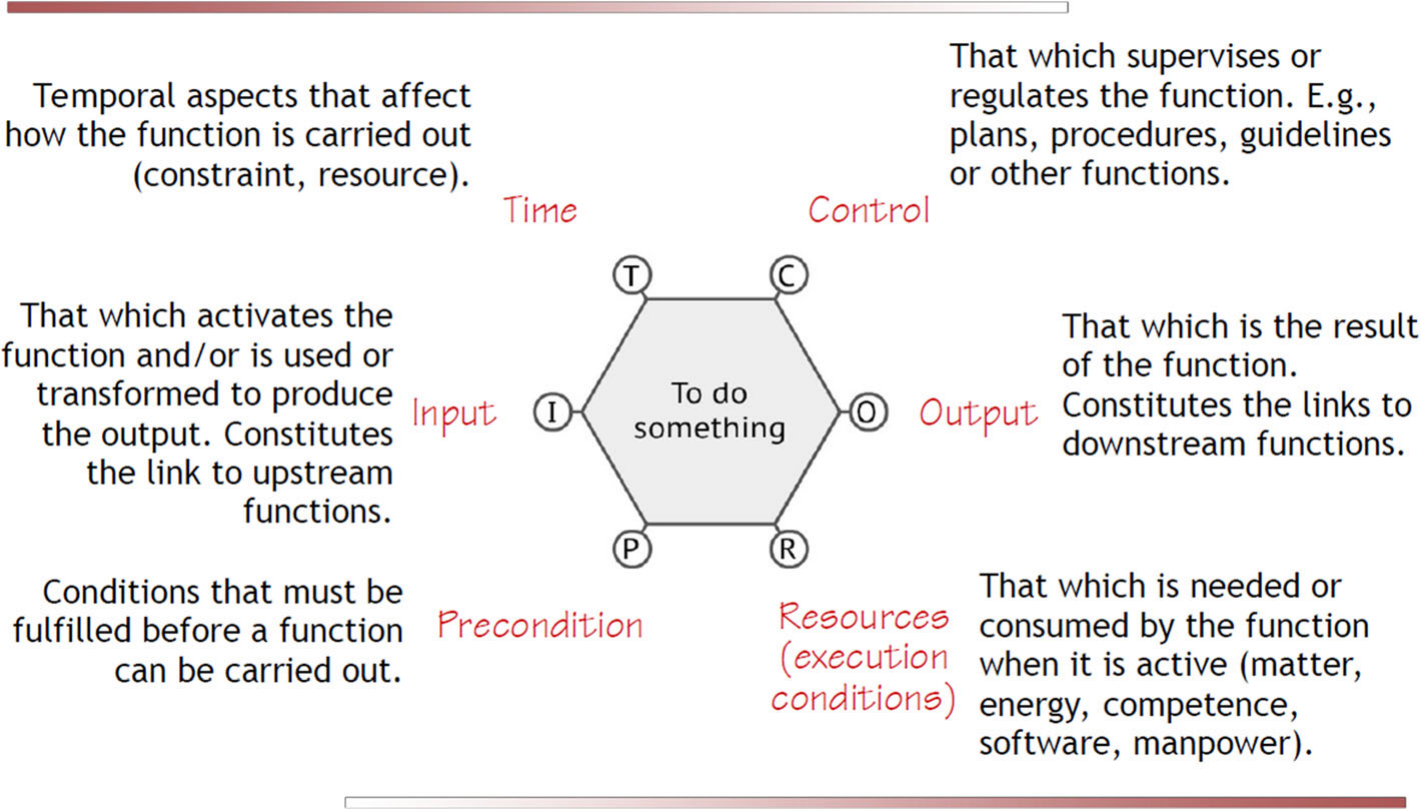
An example of a FRAM function

An approach developed outside of healthcare to analyse how a system or activity is affected by human variability is the functional resonance analysis method (FRAM) [18]. Although it is possible to produce linear engineering drawings for extremely complicated systems (such as the Large Hadron Collider), contemplating similar representation of complex sociotechnical systems, such as healthcare activities, is clearly not possible. This is particularly true as most of the systems are, additionally, complex adaptive systems, which can adapt in response to changing demands and conditions. For similar (but actually much simpler) applications, such as software programs and computer control systems, a useful approach has been to model these systems as a collection of interacting functions. Hollnagel has extended this approach to visualise complex systems as a cloud of FRAM functions with specified interacting and interdependent “Aspects”, as shown in the following diagram (Fig. 1) [18].

Using this approach, it is possible to compare and contrast how a system actually operates with different human adaptations of practice (WAD) against how it might have been designed to operate (WAI) and observe the impact of variabilities in the different interactions on the outcomes. This is achieved by detailing the WAD and reflecting this against WAI [17]. The FRAM output provides visual models of WAD as set of coupled/ interlinked functions/human actions in a system that indicates key interactions [18].

In this paper, we introduce a novel application of the FRAM and describe how the methodology can now work in conjunction with TES to explore in-depth the human adaptations evident in healthcare simulations. The study has taken advantage of a recent enhancement of the FRAM methodology, termed the FRAM model interpreter (FMI) [19], which systematically “parses” the FRAM models (as essentially computer programs) to check for completeness and validity. The resulting FRAM model is thus seen as an ordered set of production rules. The basic principle is that each function “looks” for the conditions that may activate or “trigger” it. These conditions include the inputs, of course, but also the status of the aspects that have been defined for a function. If these aspects are present, the function is activated, and the output is generated. This output will then be detected by other (downstream) functions, which then may become activated, and so on. In this way, the activity is propagated through the model according to how the relations between functions have been specified, i.e., according to the potential couplings defined by the aspects. This option allows the analyst to step through each model rigorously, one function at a time. This allows the analyst to run through a series of variabilities (human adjustments, adaptations) and to observe the propagation of the variabilities right throughout the visualisation.

This application of FRAM thus systematically interrogates the effects of variability in the interactions of critical functions. This provides a structured analysis of the downstream effects of the “what if” phenomena of human variability. As such, we have termed this “Structured What If–FRAM”, SWI-FRAM. The aim of this paper is to guide the reader through the SWI-FRAM approach and describe the added value of exploring the consequences of human variability from a Safety-II perspective to TES.

## Methods

### Study design

In order to demonstrate the structured applicability of SWI-FRAM to in situ simulation (ISS), we present a case series exploration of empirical data from the ISS management of paediatric time critical head injuries (TCHI), using the SWI-FRAM approach. Paediatric trauma systems are recognised as complex systems and globally many ISS programs aim to improve the patient safety and outcomes of paediatric TCHI [20].

### Study setting

Data collection took place in the emergency department of ten UK hospitals. Ethics approval was sought but not deemed necessary, in accordance with UK Health Research Authority guidelines. Informed consent was obtained from all participants in this study. All data sources were de-identified and stored in accordance with UK research guidelines. This study utilised the Reporting Guidelines for Health Care Simulation Research [21].

### Development of the SWI-FRAM models

**Fig. 2:**
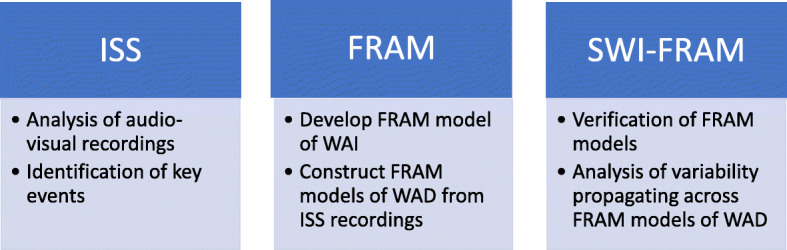
The development of SWI-FRAM models from in-situ simulations

The process for development of SWI-FRAM models from ISSs (Fig. 2) is described below.

The process had three steps:

1. Structured observation of key timings of ISSs
2. Construction of FRAM models of WAI and WAD
3. Verification of the models then visualisation of the observed variability propagation through the FRAM models of WAD.

### Structured observation of key timings of ISSs

Twenty standardised ISSs were performed in 10 UK emergency departments following an established protocol [22]. Each simulation was performed on a date and time agreed by the clinical directors of Emergency Medicine, Surgery, Anaesthesia and Nursing at each site. For the simulations, each hospital was free to organise extra staff to cover the participating trauma teams. Each hospital provided the normal representative membership of the trauma team (the real trauma team for that date). In order to avoid compromising care or actual patients, there was prior agreement to not start, or to immediately terminate, any simulation if there was a clinical need for the emergency bay or the resuscitation team. Each simulation and debrief lasted 30 min in total. The fully immersive simulation for the management of a 7-year-old child with a TCHI was created by a consensus panel of the INSPIRE Trauma Group (see online supplementary file 1). This study used was a Gaumard Hal patient simulator (Gaumard Scientific, FL, USA).

Video recordings of each simulation were independently reviewed by two researchers (RM, CK). Each video was observed for standardised key timing events deemed likely to impact patient outcomes agreed by a consensus panel of experts (INSPIRE Trauma group) [23].

The observed key events and timings in the 20 simulations are shown in Table 1, timings are displayed in minutes and seconds, ‘X’ denotes when a key event did not occur. In this series, a neurological examination (assessment of GCS and pupil check) occurred in 18 cases. A problem declaration to the trauma team of a head injury occurred in 16 cases. Only in one case was the time critical nature declared. The time (minutes:seconds) to head injury declaration, ranged from 3:56 to 20:16. Intubation occurred in 18 cases and other neuroprotective measures occurred in 9 cases.

**Table 1:** Observed key events and timings in in situ simulations

No.	Trauma Team Leader Review	Pupil check time (min: seconds)	Dilated pupil declared time	Head Injury declared time	Intubation time	Phone major trauma centre	Neuroprotection time and event
1	On arrival	6:30	6:45	6:47	16:45	18:06	17:39 Ventilate to normocapnia
2	On arrival	X	X	3:56	19:08	X	X
3	On arrival	8:35	8:40	12:31	15:06	9:15	06:34 Raise head 30°
4	On arrival	3:56	4:00	5:44	21:40	X	X
5	On arrival	3:59	4:22	5:18	30:06	18:02	X
6	01:53	9:58	10:11	X	22:20	14:40	X
7	On arrival	X	X	X	X	11:03	X
8	On arrival	3:50	4:15	4:30	18:30	6:00	11:50 Mannitol
9	On arrival	4:50	4:59	5:20	17:33	6:30	12:50 Mannitol
10	On arrival	4:00	4:20	8:45	19:01	9:30	X
11	On arrival	6:11	7:49	X	X	18:22	X
12	On arrival	3:59	4:50	6:23	16:05	X	10:35 Raise head 30°
13	On arrival	9:15	9:19	9:45	15:58	9:15	13:15 Mannitol
14	On arrival	5:07	5:25	15:40	20:31	11:00	09:30 Raise head 30°
15	On arrival	4:18	4:58	5:23: (6:33 time critical declared and repeated at 11:20)	13:47	8:30	14:00 Raise head 30°
16	On arrival	4:40	4:44	20:16	23:56	6:55	X
17	On arrival	5:50	6:30	7:55	16:15	8:40	X
18	On arrival	4:53	5:10	5.57	18:00	8:05	X
19	On arrival	2:10	9:20	9:20	18:20	11:25	14:30 Reduce venous pressure
20	On arrival	8:45	09:05	9:20	18:35	X	X

### Construction of FRAM models of WAI and WAD

**Fig. 3:**
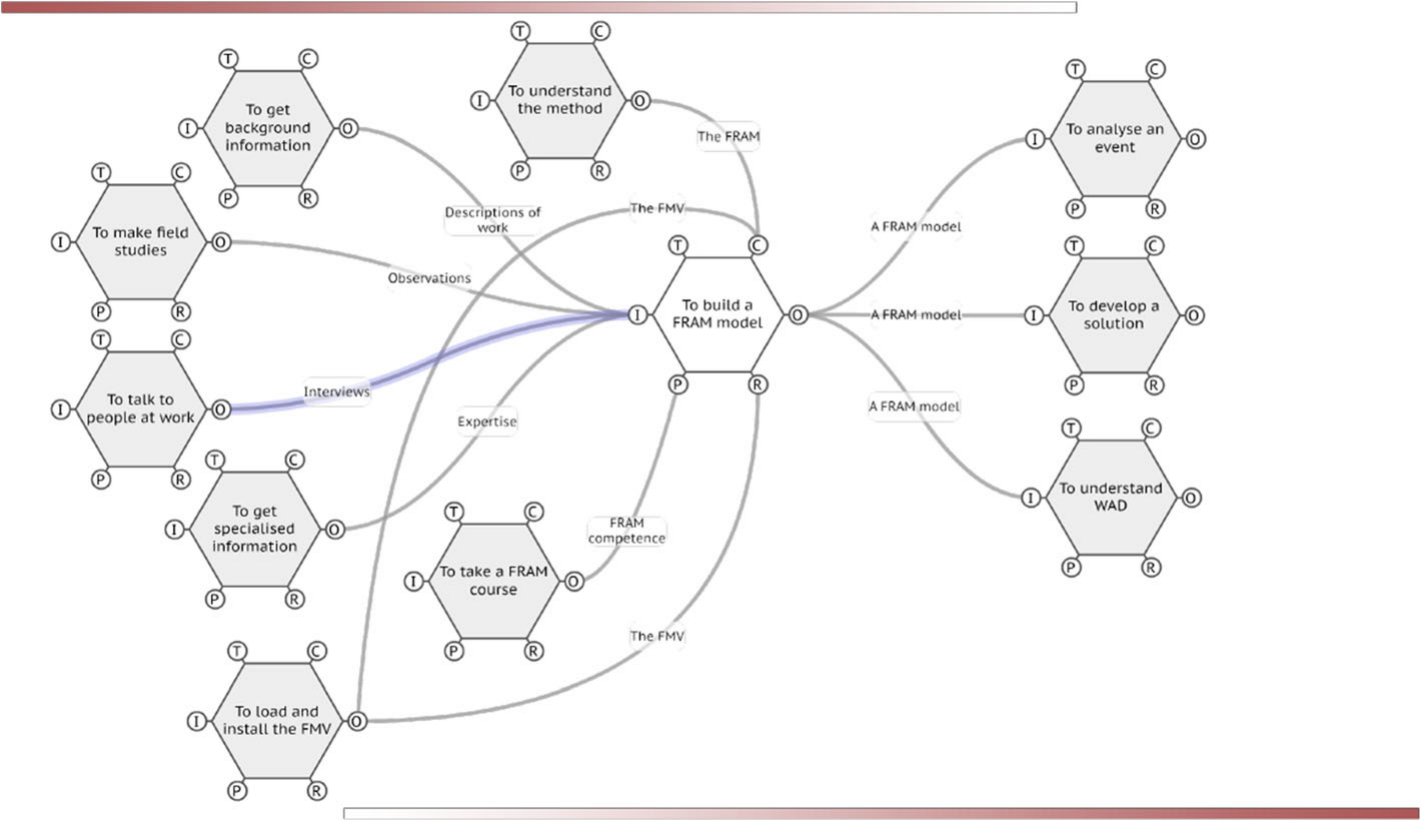
A FRAM model depicting the interacting functions involved in constructing a FRAM model. This is a relatively simple FRAM model where all functions can be easily checked in an external peer review process, then by the FMI software

A full description of FRAM is outside the remit of this paper, the reader is directed to a short introduction to the ideas and steps in FRAM modelling (supplementary file 2) and a complete online resource [24]. FRAM modelling is a sequence of logical steps of collecting data on a complex system and developing an understanding of the relationships between functions across the system. Using a software tool, the FRAM Model Visualiser, (FMV) [25], the functions are set out in an interacting, interdependent picture, as a “cloud” visualisation (Fig. 3). Each function is defined by the FRAM aspects described above (Fig. 1). In a sequence of tasks in a particular “snapshot” of the system’s operations, those functions which have to deliver before others can begin are called “upstream functions” and the subsequent functions are obviously “downstream functions” (Fig. 3). FRAM models are validated externally by expert opinion to ensure they accurately reflect WAI or WAD.

### Construction of the FRAM model of WAI for the management of TCHI

Two researchers familiar with FRAM (RM, DS) created the FRAM model of WAI, using the same evidence based guidelines for the management of paediatric TCHI, as used in the construction of the standardised ISS scenario.

Initially, a FRAM model of WAI of all the functions involved in the TCHI management was constructed (see online supplementary file 3). Then, a simplified FRAM model of WAI was constructed that focused on a specific area of interest, the functions (steps in the care process), linked to the observed key timings. In this manuscript, the focus of attention is on the identification of a TCHI during the initial primary survey assessment of the child, the declaration of this problem to the team and the commencement of management to prevent secondary brain injury and transportation for neurosurgical input. Focus of attention may be placed upon any area in the system of choice and FRAM models can be created to explore the variations and adaptations accordingly.

### Construction of FRAM models of WAD

The two above researchers reviewed the 20 video recordings and created FRAM models of WAD for each of the ISSs, focusing upon the chosen area of interest described above.

### Structural verification of the FRAM models

#### Expert validation and internal checking of the FRAM models

The models generated were independently checked by two other authors (KPH, CK) both subject matter experts in simulation and management of paediatric TCHI. During these sessions, the research team members who did not create the FRAM models had opportunity to verify that the FRAMs captured the critical elements of the management of TCHI that was of interest (examination of neurological status and subsequent actions).

The FMI software [19] was used to internally check the structural integrity of the FRAM models (WAI and WAD) for consistency and completeness and that the endpoints could be reached as intended.

#### Visualisation of variability propagation through FRAM models

The FMI software [19] was then used to explore what effects the variabilities in the outputs of each function in turn have on the downstream functions’ operation. Based on the ISSs, the variability of each function was coded in terms of time (early, on time, too late, not at all) or precision (precise, acceptable or imprecise) allowing the “what if” analysis of the impact of the variabilities on the functions leading to the model output.

The observed variability of management of TCHI in the ISS recordings are presented as a case series of FRAM models of WAD. The FRAM models of WAD selected in the case series each depict a different adaptation from the FRAM model of WAI, evident by observing the ISS recordings.

## Results

In this section, we present the FRAM model of WAI and the simplified version. We also present the observed variability of management of TCHI in the ISS recordings, as a case series of FRAM models of WAD. The FRAM models of WAD selected in the case series each depict a different adaptation from the FRAM model of WAI, evident by observing the ISS recordings. We then show how the SWI-FRAM approach provides the opportunity to visualise the consequences of variability around key functions, as the effects propagate through the system. In order to do so, the reader is directed to the videos provided in supplementary online files 4, 5 and 6.

### The FRAM model of WAI

**Fig. 4:**
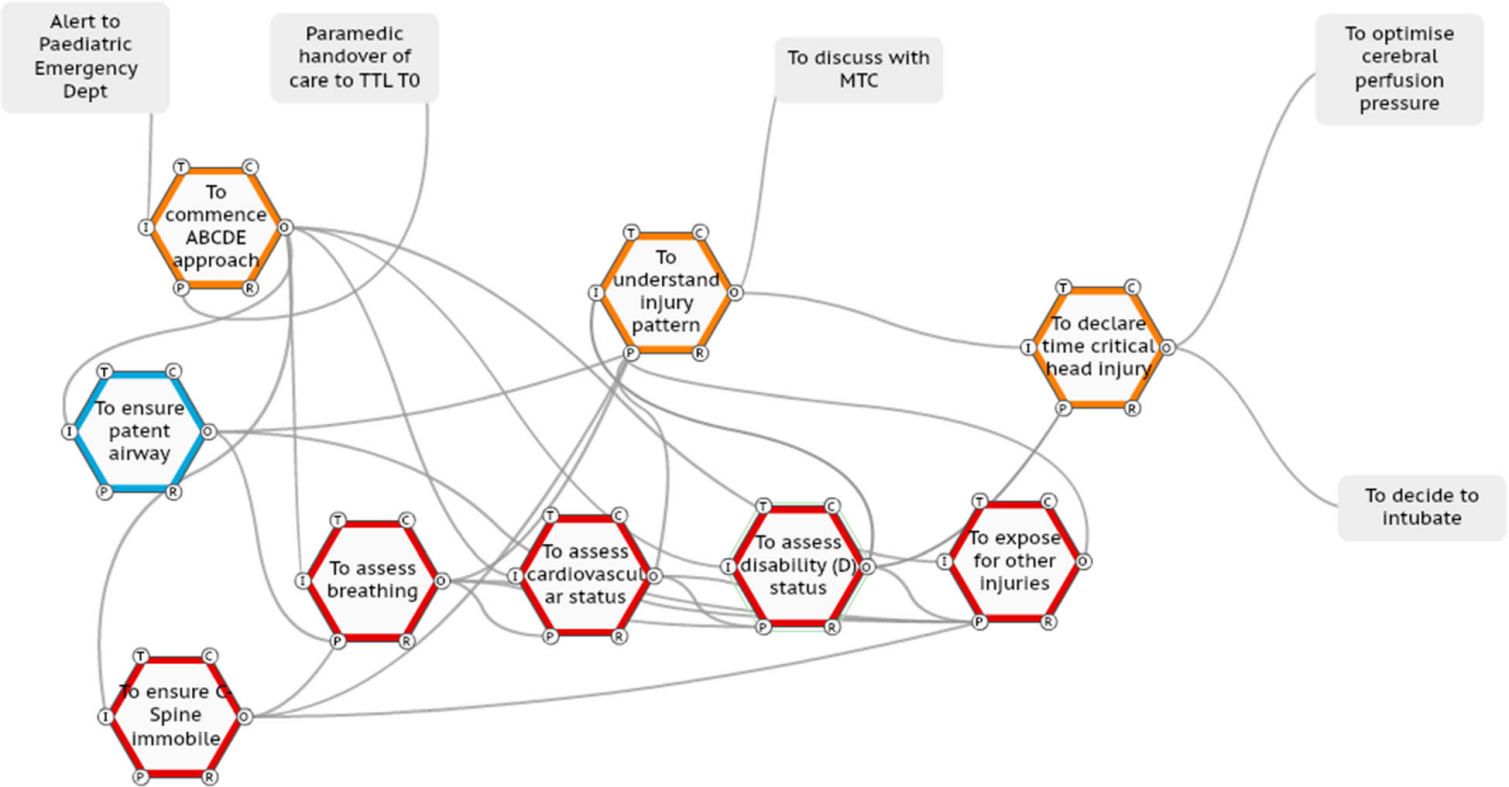
The FRAM model of WAI

The focused FRAM model of WAI for the ISS management of a standardised scenario of a 7-year-old child presenting with evolving signs of raised intracranial pressure, a unilateral fixed dilated pupil and a depressed level of consciousness, following a road traffic accident (RTA) is shown in Fig. 4.

In this FRAM model, the functions performed by key team members are identified by different colouration; orange is the trauma team leader (TTL), red is the emergency department specialist trainee doctor, and blue is the anaesthetic doctor.

In this model, all the functions in this simplified version of the WAI management of a paediatric TCHI and how they are coupled can be observed. The inter-relationship of the components of the primary survey, namely the airway, breathing, cardiovascular, disability / neurological assessment and exposure (ABCDE) assessment can be visualised. The entire FRAM model of WAI is available online (supplementary file 3).

### The case series of FRAM models of WAD

#### Case one

**Fig. 5:**
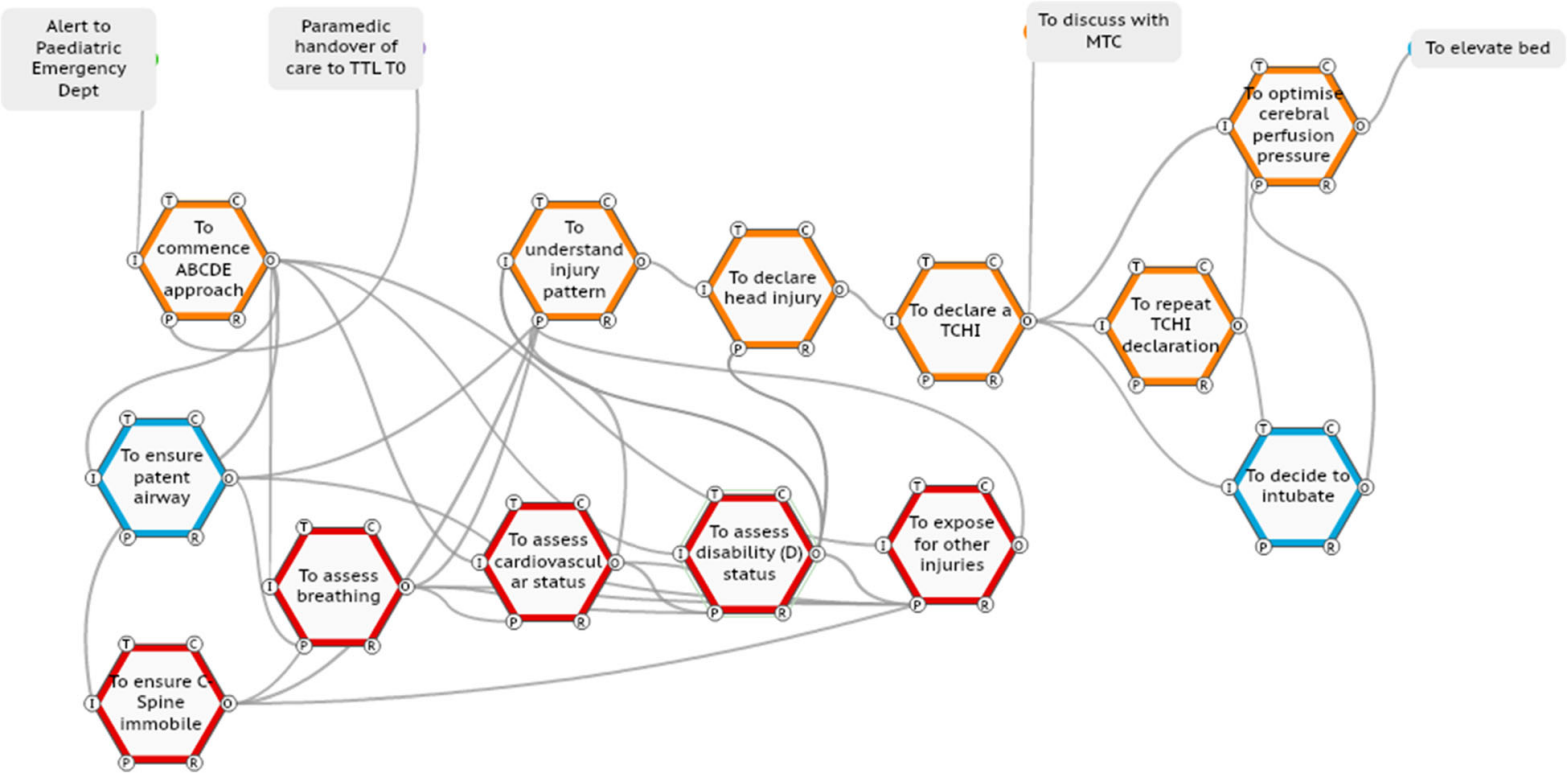
TTL adaptation of declaration of time-critical head injury to the team

In this case (ISS number 15), the TTL (orange functions) performs the primary survey as per WAI to determine the injury pattern and necessary immediate actions and declares to the team the presence of a head injury (Fig. 5). The TTL then goes further and declares to the team (70 seconds later) that this is a time critical head injury. No other TTL in the series of 20 ISSs problem declares a time critical head injury. The TTL then repeats this declaration 4 minutes later; this expediates the intubation process and ensures the patient’s bed head is elevated to enhance neuro-protection.

#### Case two

**Fig. 6:**
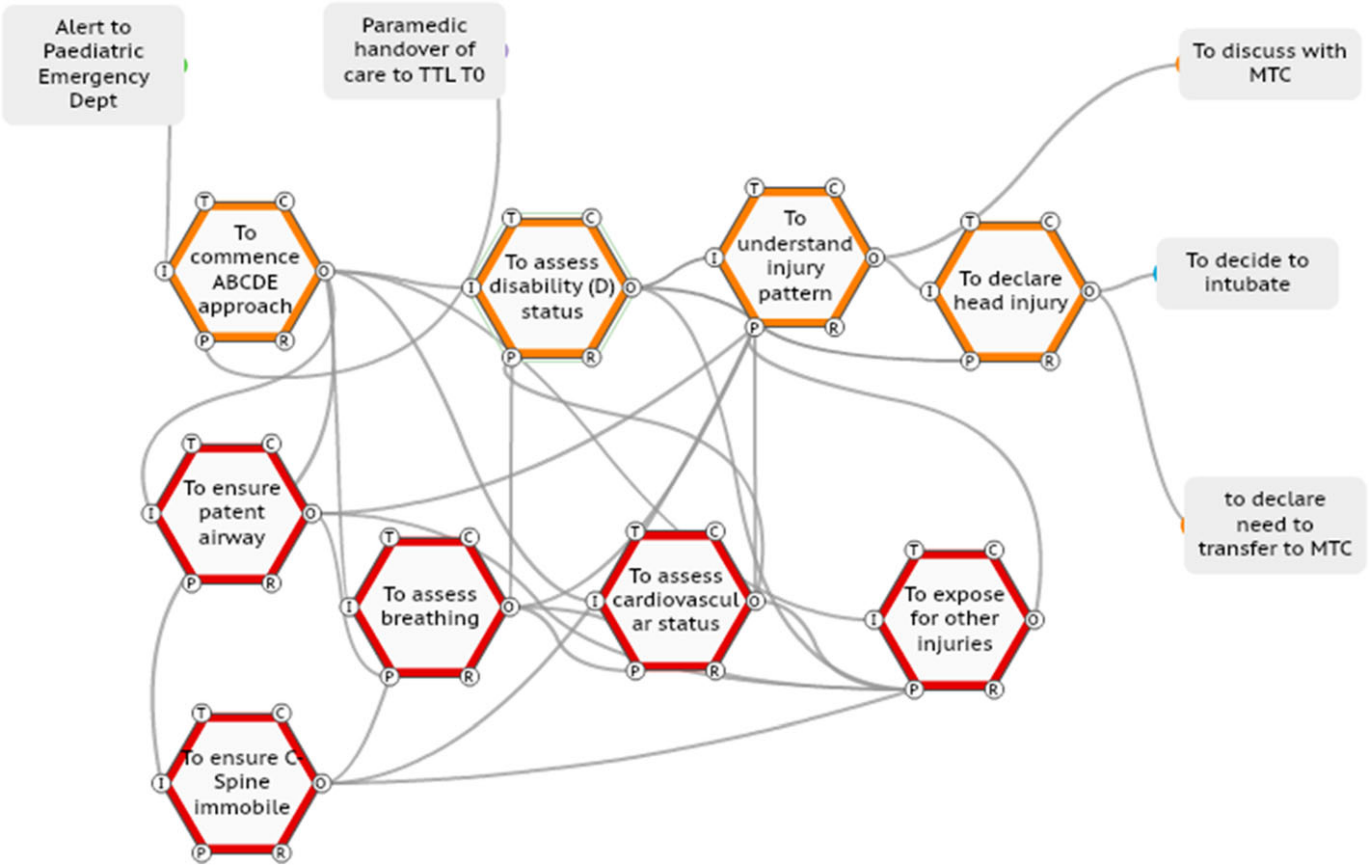
TTL adaptation of assessing the neurological status of the child then declaring head injury to the team

In this case (ISS number 5), the TTL coordinates the primary survey but decides to assess the neurological status of the child herself. This function is normally performed by a specialist trainee, with the TTL standing back. The primary survey is completed, the injury pattern determined and the TTL declares to the team the presence of a head injury (Fig. 6), the care is expediated and the end points are successfully met.

#### Case three

**Fig. 7:**
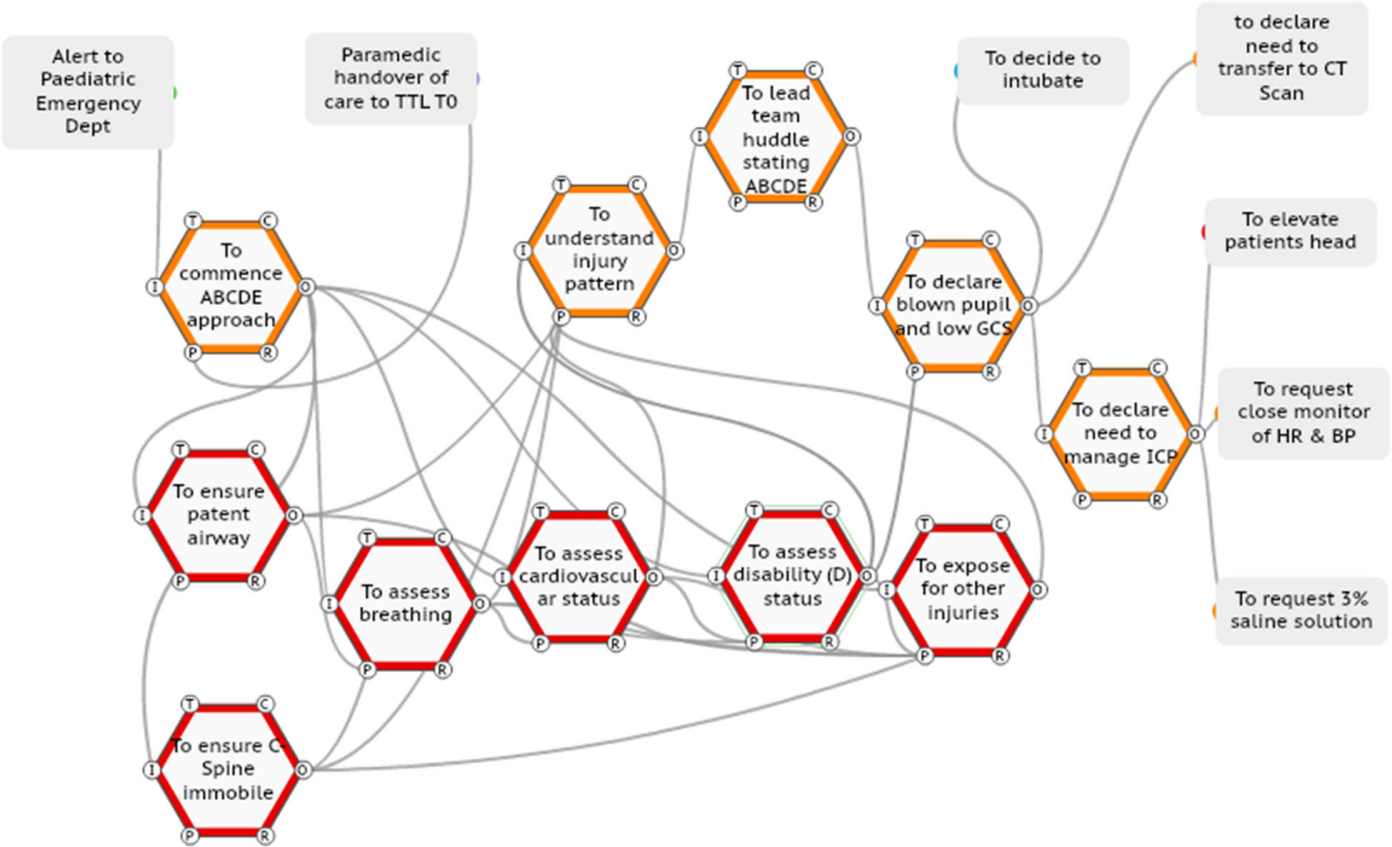
TTL adaptation of leading a brief team huddle then declaring findings and actions required to the team.

In this case (ISS number 12), the TTL coordinates the primary survey and leads a team huddle with the team and states aloud the ABCDE findings. The TTL then declares to the team the presence of a blown pupil, decides to intubate and declares the need to manage intracranial pressure issues (Fig. 7). This adaptation also expediated the care process.

#### Case four

**Fig. 8:**
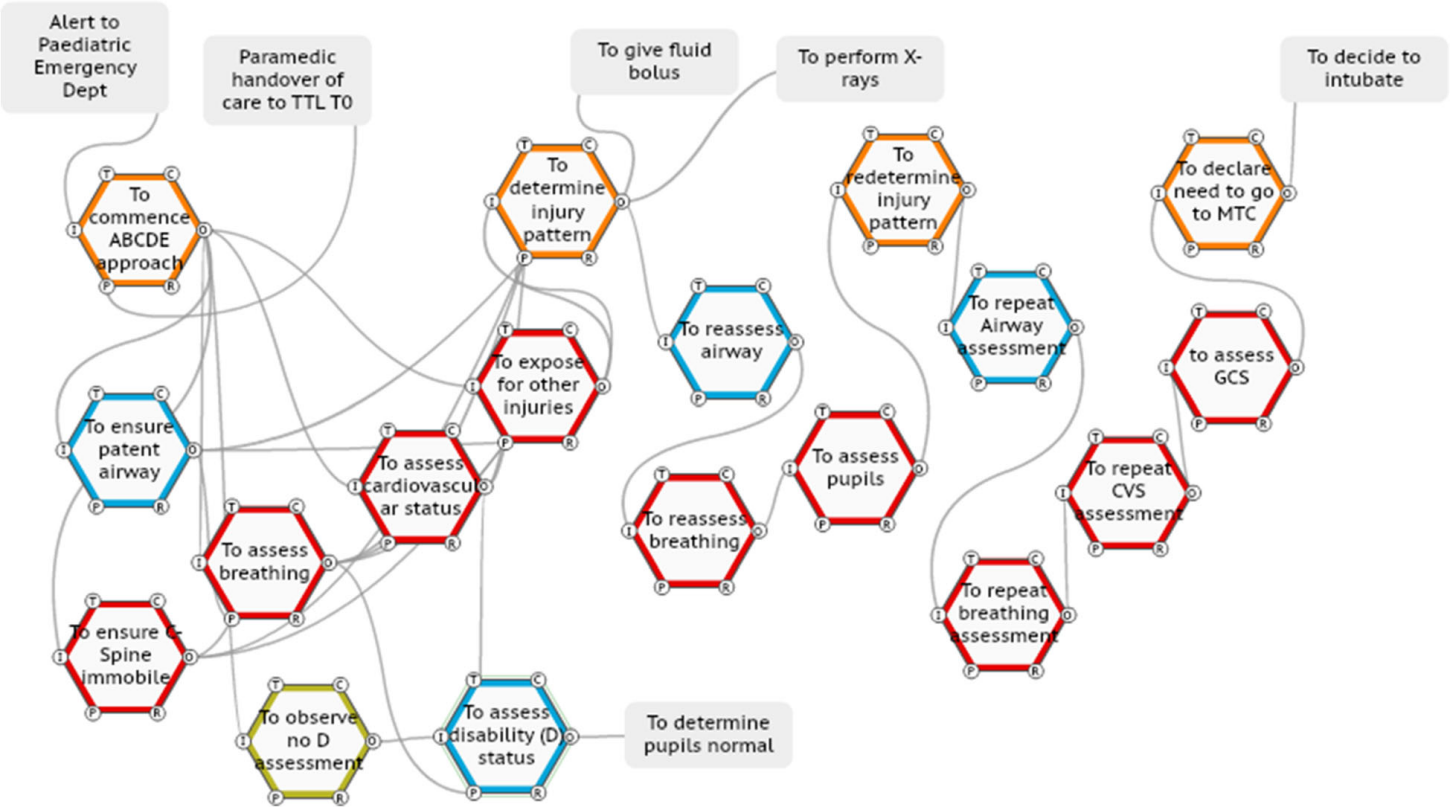
TTL adaptation of continual reassessment to determine injury pattern

In this ISS (number 11), the primary survey commences and the there is no initial D (disability) assessment (Fig. 8). This is observed by a nurse who articulates this to the anaesthetic doctor at the head of the bed. The nurse and doctor examine the pupil and consider this as normal (the child had a unilateral fixed dilated pupil). This information is not relayed to the TTL. There is no problem declaration by the TTL, who instead requests a further ABCDE assessment. During this cycle of the primary survey, the emergency department specialist trainee examines the pupils and determines them to be abnormal and relays this to the TTL. The TTL requests a further primary survey, the GCS is determined to be low and the TTL declares that the child needs to go for neurosurgical intervention at the major trauma centre.

### The SWI-FRAM approach

#### The verification of FRAM models

The structural integrity of all of the FRAM models was verified. To demonstrate this, the reader is directed to the online supplementary video of the FRAM models of WAI in this paper (supplementary files 4 and 5). All the functions and interconnections can be visualised in turn as the model cycles through each action.

#### Visualisation of variability propagating through a FRAM model of WAD

**Fig. 9:**
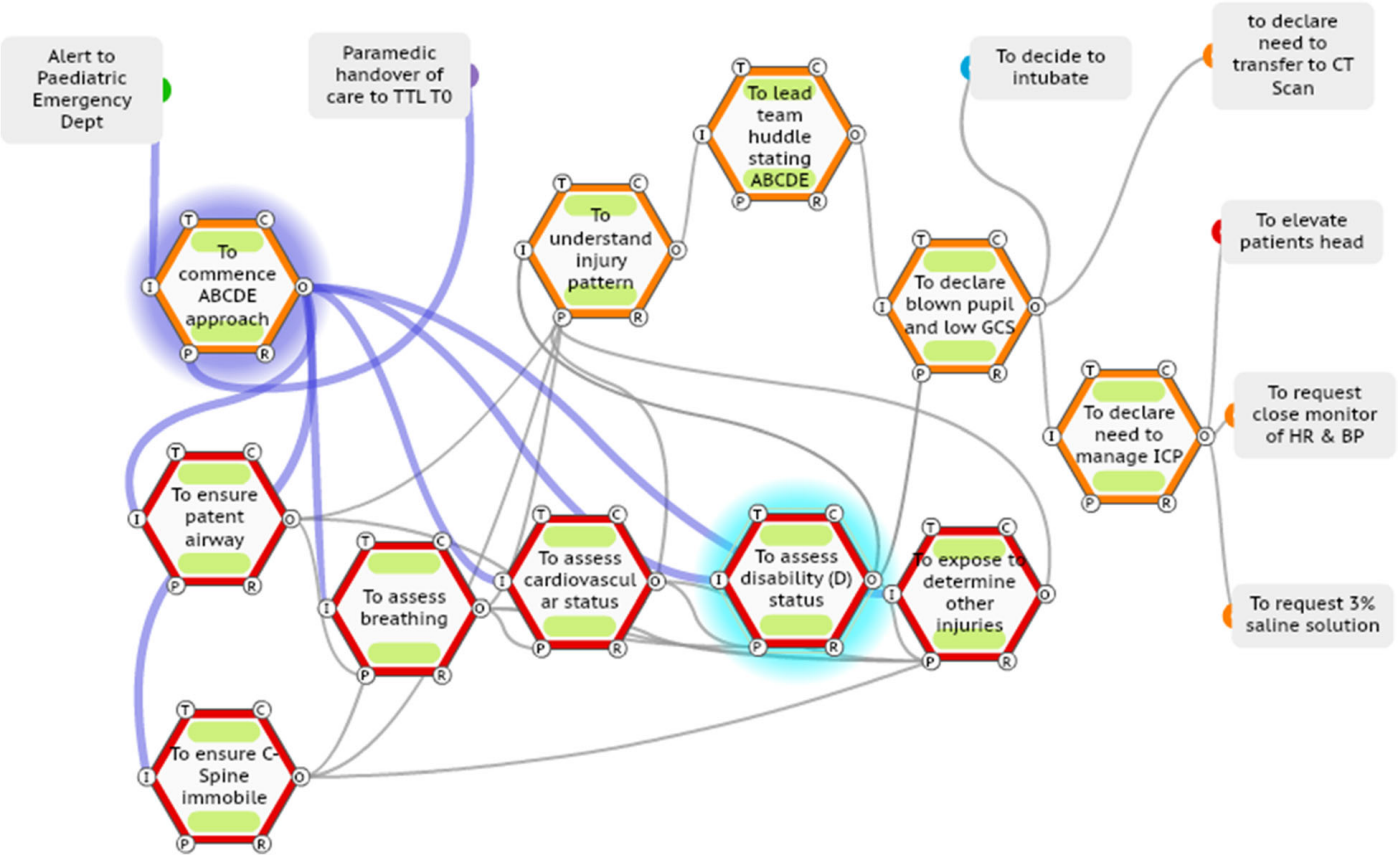
The SWI-FRAM analysis of the case three (ISS 12). In this figure, the SWI-FRAM analysis is shown cycling through the system (see online video supplementary file 6 also), as the disability / neurological assessment (checking pupils and GCS) is completed, the analysis will continue through to the huddle, then the declaration and on-going care plans

In this section, a SWI-FRAM model is presented for the third case (ISS number 12) shown in Fig. 9. A SWI-FRAM log sheet is also provided for the case. As this is a visual process, the reader is also directed the online resource (supplementary file 6).

By observing SWI-FRAM models, the variability of functions in terms of precision and timing observed in the ISS cases (Table 1) can be seen to propagate across the system. The key functions that ensure the propagation are visible, as are the sequelae of the adaptations, as shown in Fig. 9.

The SWI-FRAM reveals the flow of the functions that occurs leading to the determining the pattern of injury, the problem declaration and the subsequent actions (see also the online supplementary file 6). The SWI-FRAM approach systematically allows one to visualise how the ABCDE assessments cycle to the TTL are not linear and the how the huddle facilitates a check on all of the ABCDE leading to the desired end-points of problem declaration and also the subsequent instructions to team members to neuro-protect the child. The SWI-FRAM log sheet highlights critical variabilities, consequences and provides the option for recommendations (Table 2).

**Table 2:** The SWI-FRAM log sheet for case 3 ISS number 12

SWI-FRAM LOG SHEET				
Case: ISS number 12 Entry functions: Alert to hospital of trauma case and paramedic hand-over Exit functions: Transfer to MTC				
Function	Critical variability identified	Visualised consequence	Recommendations	Priority
**To understand injury pattern**	Precise	Rapid declaration of the problem identified to the team.	Share and adopt this practice	High
**To organise a team huddle**	Precise	Articulation systematically of the key findings of the primary survey	Share and adopt this practice	High
**To declare the need to manage raised intracranial pressure**	Precise	Acceleration of team actions to neuro-protect child and facilitate onward transfer	Share and adopt this practice	High

## Discussion

This study uses a series of structured case simulations to determine variability in performance during the management of a paediatric TCHI. FRAM models are constructed of the dependencies and actions looking at information flow and task distribution. Using independently determined metrics of the performance and timing of key actions during the scenarios from video analysis, the ideal and suboptimal performances of 20 teams were assessed and FRAM models were constructed. The human variability evident in the different simulations, in terms of different adaptations used to achieve the goals of managing the TCHI scenario, were explored using the SWI-FRAM approach.

Technology enhanced simulation has been used to explore the complexities of the interactions between humans, teams and organisations for over two decades [2]. The SWI-FRAM approach as described in this paper is a first step to illuminating how to systematically analyse variations in practice and observe the consequences of these human adaptations (both positive and negative) within simulated complex systems. The use of ISS data to create FRAM models is novel. Previous data collection in healthcare FRAMs have used interviews, focus groups and document analyses including accident investigations [26].

With investment be that time, effort and resources, there is the expectation of a return. So, what is the added value of the SWI-FRAM technique on top of current system focused simulations and system focused debriefing? The answer lies within the visual depiction of the simulated system of choice provided by the SWI-FRAM models, which can develop an understanding of how adaptations and changes can resonate through the system and lead to changes in safety or performance. The models provide (1) an holistic overview, (2) a depth of understanding of critical functions within the system, (3) a visualisation of non-linear interrelationships between functions, (4) the ability to observe the downstream effects of the variability of upstream functions inherent in different simulations, (5) the opportunity to discuss and develop interventions that embrace the complexity to enhance the safety and performance of the system explored. In addition to the visual models, the log report output provided by the software also provides information in a format that speaks to organisational learning goals.

The holistic overview provided by SWI-FRAM models includes the ability to systematically step through every part of a complex system visually, for example the FRAM model of WAI, (online supplementary file 4) and the ability to observe downstream consequences of upstream variations in practice, as described in the case series. This overview can be utilised to create an awareness of the complexity at the individual, team and organisational level, this includes enhancing an appreciation of the differing roles of individuals and how they interact. This may be a strategy to enhance current deficits in the sharing of mental models across team members, for example that particularly evident in managing paediatric TCHI [27]. The critical functions within the system can be analysed in depth, in this study a focus was placed upon the neurological assessment to identify a low GCS score and a fixed and dilated pupil, then the problem declaration by the trauma team leader. The SWI-FRAM approach makes it possible to distil down processes and focus upon key interactions in the system as shown in the case series of FRAM models of WAD (Figs. 5, 6, 7, 8, and 9). The human adaptations highlighted in the case series, in terms of problem declaring the time critical nature of the injury, the decision as TTL to perform the neurological assessment, the team huddle and the continual rechecking can be clearly visualised. In addition to verifying the internal consistency of each model the SWI-FRAM approach then allows the effects of the variable adaptations to be seen propagating through the models to their end points (Fig. 9 and online supplementary file 6). This can then be used to develop interventions or strategies to cope with the complexity and then embed them into systems [28]. One example from the case series presented could be the development of the scribe role to directly support a TTL. In ISS case 11 (Fig. 8), the nurse observes the absence of the neurological assessment in the primary survey and approaches the anaesthetic doctor to perform the assessment together, without bringing the TTL into this loop of functions. The addition of an additional function “to ensure all assessments are reported to the TTL” by an experienced person in the role of the scribe may enhance safety and the performance in this system.

The SWI-FRAM approach facilitates the direct visualisation of the normal mundane adaptations that occur in everyday practice to enhance patient safety. In doing so, it has the potential to focus reflection on action in the real world of everyday clinical practice to deeply analyse what goes well most of the time and why, what trade-offs occur and what are the consequences. There is the ability with SWI-FRAM to observe what happens when an expert or a novice in a given context with multiple options, chooses one and how resultant changes resonate across the system. There is the ability to observe adaptations to different contexts, in terms of individual’s competencies, process standards/guidelines and differing material environments that occur in real life or can be created in different simulations [15]. As such the SWI-FRAM approach may influence scenario designs. By providing insights on what works in given contexts, the SWI-FRAM approach may also be useful to debriefing frameworks that aim to promote second order learning that focuses upon how to generalise and learn to adapt to new situations [15].

One more return on investment for performing the SWI-FRAM approach may be to enhance further the influence that TES may have on the senior hospital management to invoke changes to improve patient outcomes. The visual output, including potential videos of propagating effects of simulated changes in systems and the report log may provide another readily comprehensible stimulus, in a format that can link with knowledge management frameworks [29] and facilitates organisational learning [30].

### Limitations

This SWI-FRAM approach presented is anchored in ISSs that are considered “as close” to real-life as possible. As such the system based simulations described remain proxy considerations to real-life clinical care. In this study, a number of steps were taken to minimise bias and enhance transparency, including standardised simulations and independent rating of the simulations, the use of a clinical expert and a non-clinical patient safety expert as the initial FRAM analysts and then a second team of trauma care subject matter experts to independently iteratively validate each model. Reflexivity was maintained by continual dialogue between the international group of researchers. Expertise is required to develop and analyse the FRAM models as described in this manuscript; this expertise is becoming increasingly available within organisations that already perform ISSs.

## Conclusions

TES as a tool that can safely replicate almost any patient—healthcare professional/team—system interaction, is well placed to explore human performance adjustments and performance variability. As such, there is the opportunity to consider both Safety-I and Safety-II perspectives to enhance patient safety and patient outcomes. By focusing upon and visually depicting the consequences of human variability, the SWI-FRAM approach provides a holistic overview of the extent of complexity, a visualisation of non-linear interrelationships between critical functions and an exploration of what goes right, in addition to what goes wrong in a system. The novel and effective basis of this approach is that it concentrates on the functions essential to ensure successful outcomes, without necessarily being constrained by identifying individual agents, human or organisational. This focus on functions thus clarifies and simplifies the analysis and is not distracted by the inevitable difficulty of discerning exactly what roles or agents are performing the functions in the crowded intensive work-spaces encountered in real life. In doing so, this approach enhances the opportunity to discuss and develop more effective interventions to enhance the safety and performance of the simulated system explored, that can proactively impact future real-life situations.

## Supplementary Information

**Additional file 1.** The standardised in-situ simulation scenario design.

**Additional file 2.** The construction of FRAM models of work-as-imagined (WAI) and work-as-done (WAD) and the SWI-FRAM approach.

**Additional file 3.** WAI.

**Additional file 4.** WAI CAS.

**Additional file 5.** focused WAI.

**Additional file 6.** SWI-FRAM WAD.

## Data Availability

Please contact the author for data requests. Only anonymised data will be available.

## Abbreviations

TES: Technology enhanced simulation
ISS: In situ simulation
WAI: Work-as-imagined
WAD: Work-as-done
FRAM: Functional resonance analysis method
SWI-FRAM: Structured what if–FRAM
TCHI: Time critical head injuries
INSPIRE: International Network for Simulation-based Pediatric Innovation, Research, and Education
FMI: FRAM model interpreter
FMV: FRAM model visualizer
RTA: Road traffic accident
TTL: Trauma team leader
ABCDE: Airway, breathing, circulation, disability, exposure

## References

[1] Kolb DA. Experiential Learning : Experience as the Source of Learning and Development. Englewood Cliffs, NJ: Prentice-Hall.; 1984.

[2] Rosen AM, Hunt AE, Pronovost JP, Federowicz AM, Weaver JS. In Situ Simulation in Continuing Education for the Health Care Professions: A Systematic Review. J Contin Educ Health Prof. 2012;32:243–54. 10.1002/chp.21152 23280527

[3] Gaba DM, Howard SK, Fish KJ, Smith BE, Sowb YA. Simulation-Based Training in Anesthesia Crisis Resource Management (ACRM): A Decade of Experience. Simul. Gaming. 2001;32:175–93.

[4] Hollnagel E. Coping with complexity: past, present and future. Cogn Technol Work. 2012;14:199–205.

[5] Flach J. Complexity: learning to muddle through. Cogn Technol Work. 2012;14:187–97.

[6] Hollnagel E WR, Braithwaite J. From Safety-I to Safety -II: A white paper. 2015. https://www.england.nhs.uk/signuptosafety/wpcontent/uploads/sites/16/2015/10/safety-1-safety-2-whte-papr.pdf. Accessed 18 May 2020.

[7] Reid JMD, Stone KMD, Huang LMD, S. Deutsch EMD. Simulation for Systems Integration in Pediatric Emergency Medicine. Clin. Pediatr. Emerg. Med. 2016;17:193–9.

[8] Dubé MM, Reid J, Kaba A, Cheng A, Eppich W, Grant V, et al. PEARLS for Systems Integration: A Modified PEARLS Framework for Debriefing Systems-Focused Simulations. Simul Healthc. 2019;14:333–42. 10.1097/SIH.0000000000000381 31135684

[9] Holden RJ, Carayon P, Gurses AP, Hoonakker P, Hundt AS, Ozok AA, et al. SEIPS 2.0: a human factors framework for studying and improving the work of healthcare professionals and patients. Ergonomics. 2013;56:1669–86. 10.1080/00140139.2013.838643 PMC3835697 24088063

[10] Adler MD, Mobley BL, Eppich WJ, Lappe M, Green M, Mangold K. Use of simulation to test systems and prepare staff for a new hospital transition. J Patient Saf. 2018;14(3):143–7. 10.1097/PTS.0000000000000184 26076076

[11] Ventre KM, Barry JS, Davis D, Baiamonte AAVL, Wentworth AC, Pietras M, et al. Using In Situ Simulation to Evaluate Operational Readiness of a Children’s Hospital-Based Obstetrics Unit. Simul Healthc. 2014;9:102–11. 10.1097/SIH.0000000000000005 24401917

[12] Patterson MD, Geis GL, Falcone RA, LeMaster T, Wears RL. In situ simulation: Detection of safety threats and teamwork training in a high risk emergency department. BMJ Qual Saf. 2013;22:468–77. 10.1136/bmjqs-2012-000942 23258390

[13] Geis GL, Pio B, Pendergrass TL, Moyer MR, Patterson MD. Simulation to Assess the Safety of New Healthcare Teams and New Facilities. Simul Healthc. 2011;6:125–33. 10.1097/SIH.0b013e31820dff30 21383646

[14] Fanning RM, Gaba DM. The role of debriefing in simulation-based learning. Simul Healthc. 2007;2(2):115–25. 10.1097/SIH.0b013e3180315539 19088616

[15] Dieckmann P, Patterson M, Lahlou S, Mesman J, Nyström P, Krage R. Variation and adaptation: learning from success in patient safety-oriented simulation training. Adv Simul. (London). 2017;2(1):21. 10.1186/s41077-017-0054-1 PMC5806267 29450022

[16] Wears R, Hollnagel E, Braithwaite J. The resilience of everyday clinical work. London: Ashgate Publishing Ltd.; 2015.

[17] Braithwaite J, Wears RL, Hollnagel E, editors. Resilient health care: volume 3 : reconciling work-as-imagined and work-as-done. Boca Raton, FL: CRC Press, Taylor & Francis Group USA, 2017.

[18] Hollnagel E. FRAM: The functional resonance analysis method, modeling complex socio-technical systems. London: Ashgate Publishing Ltd.; 2013. p.117–8.

[19] Hollnagel E. The FRAM Model Interpreter. 2020. https://functionalresonance.com/the-fram-model-interpreter.html. Accessed 7 Nov 2020.

[20] Jensen AR, McLaughlin C, Wong CF, McAuliff K, Nathens AB, Barin E, et al. Simulation-based training for trauma resuscitation among ACS TQIP Pediatric centers: Understanding prevalence of use, associated center characteristics, training factors, and implementation barriers. Am J Surg. 2019;217:180–5. 10.1016/j.amjsurg.2018.06.009 PMC7169990 29934123

[21] Cheng A, Kessler D, MacKinnon R, Chang TP, Nadkarni VM, Hunt EA, et al. Reporting guidelines for health care simulation research: extensions to the CONSORT and STROBE statements. (Report). Adv Simul. 2016;1(1). 10.1186/s41077-016-0025-y PMC5806464 29449994

[22] MacKinnon RJ, Kennedy C, Doherty C, Shepherd M, Cole J, Stenfors-Hayes T. Fitness for purpose study of the Field Assessment Conditioning Tool (FACT): a research protocol. BMJ Open. 2015;5:e006386. 10.1136/bmjopen-2014-006386 PMC4401849 25869682

[23] The International Network for Simulation-based Pediatric Innovation Research & Education (INSPIRE). https://www.inspiresim.com. Accessed 23Feb 2021.

[24] Hollnagel E. FRAMILY. 2020. https://functionalresonance.com/framily-meetings/framily%202018-2/framily%202018%20tutorial%20resources.html. Accessed 18 May 2020.

[25] Rees C. FRAM Model Visualiser. 2020. http://zerprize.co.nz/FRAM/index.html. Accessed 18 May 2020.

[26] Patriarca R, Di Gravio G, Woltjer R, Costantino F, Praetorius G, Ferreira P, et al. Framing the FRAM: A literature review on the functional resonance analysis method. Saf. Sci. 2020;129:104827.

[27] Auerbach M, Cole J, Violano P, Roney L, Doherty C, Shepherd M, et al. An International Interprofessional Study of Mental Models and Factors Delaying Neuroimaging of Critically Head-Injured Children Presenting to Emergency Departments. Pediatr Emerg Care. 2018;34:797–801. 10.1097/PEC.0000000000000915 27753711

[28] Macrae C. Moments of Resilience: Time, Space and the Organisation of Safety in Complex Sociotechnical Systems. In: Wiig S, Fahlbruch B, editors. Exploring Resilience: A Scientific Journey from Practice to Theory. Cham: Springer International Publishing; 2019. p. 15–23.

[29] Salzano KA, Maurer CA, Wyvratt JM, Stewart T, Peck J, Rygiel B, et al. A Knowledge Management Framework and Approach for Clinical Development. Ther Innov Regul Sci. 2016;50:536–45. 10.1177/2168479016664773 30231759

[30] Cox M, Irby DM, Reznick RK, MacRae H. Teaching Surgical Skills — Changes in the Wind. New Engl J Med. 2006;355:2664–9. 10.1056/NEJMra054785 17182991

