# Supplementary material for: A novel approach to explore Safety-I and Safety-II perspectives in in situ simulations—the structured what if functional resonance analysis methodology
Source: Adv Simul (Lond). 2021 Jun 5;6:21. doi: 10.1186/s41077-021-00166-0 (PMC8178899; doi:10.1186/s41077-021-00166-0)
Supplement: Supplementary file 1 — Additional file 1. The standardised in-situ simulation scenario design. [file 41077_2021_166_MOESM1_ESM.docx]

# SUPPLEMENTARY FILE 1. THE STANDARDISED IN-SITU SIMULATION SCENARIO DESIGN

1. Timings & Veto of Simulations

2. Setting

3. Equipment safety

4. Position of cameras

5. Overall Goals

6. Learning Objectives

7. Faculty Requirement
8. Participants
9. Scenario details
10. Patient Demographics

11. Equipment requirement

12. Standardised time-line

13. Expected progression of simulation

14. Debriefing key points

**1. Timings & Veto of Simulations**

All dates and timings were agreed with department heads at the trauma units.

The simulations were conducted at 0900.

During the development phase at the Major Trauma Centre, it was agreed that the simulations would run in the mornings. The simulations only ran on weekdays.

Simulations were not permitted to run if there was a likelihood that care of other patients would be negatively impacted.

Any member of the nursing or medical team could veto a simulation running at any point.

In the event of a real child arriving, it was agreed that the simulator would be immediately removed and the trauma team performing the simulation would remain and manage the child.

**2. Setting**
All scenarios were run in Emergency Department (ED) Resuscitation Bays.

**3. Equipment safety**

In order to prevent any contamination of simulation equipment, all equipment used was ED equipment.

The simulator had a neck collar on and a 22G cannula attached to the left tibia, oxygen mask and tubing, ECG, BP & oxygen saturation leads and a spinal board from the ED stock were in place. These remained with the simulator and would be replenished from ED stock if removed and discarded by staff.

The cited information regarding the patient is not an actual patient. Any resemblance to real person living or deceased will be coincidence. 

**4. Position of cameras**

One camera was situated on the wall behind the resuscitation bed. Another camera was situated at 45 degrees to the bed on the wall/stand.

**5. Overall Goal**

To provide real-time inter-professional training in the management of the child with a time-critical head injury.

**6. Learning Objectives**

a. Demonstrate recognition and assessment of a seriously injured child.

b. Demonstrate knowledge and management of a time-critical head injury

c. Recognise and respond to signs of raised ICP

d. Demonstrate knowledge of the further communication and preparation required for definitive care of a time-critical head-injured child

e. Demonstrate effective team working in emergency bay

**7. Faculty Requirement**
Number: 3
Specific roles

i). Facilitator/Confederate

ii). Audio-visual operator

iii). Analyser

**8. Participants**Expected number: 7
Specific roles:

1. Trauma Team Lead (TTL)

2. Emergency Department Nurse 1

3. Emergency Department Nurse 2

4. Anaesthetist

5. Surgeon

6. Parent Liaison Nurse

7. Emergency Department Physician

**9. Scenario Details**

A 7-year-old boy ran away from his mum, stepped into the road and has been hit by a passing car which was going relatively slowly. The incident occurred 45 minutes previously and the ambulance crew arrived on the scene within 5 minutes, whereupon the boy’s observations were: GCS 6, RR 12, HR 135 & BP 85/60.

The child is brought into A&E by a paramedic on a spinal board with his neck stabilised in a hard cervical collar. He is receiving 10L/min of oxygen by face mask and has a blue cannula in left tibia (saphenous vein).

There are no available details about the child, but the police are following by car and have the child’s parent with them.

On arrival in Emergency Department he was GCS 8 (13 at scene), respiratory rate 12/minute, HR 120/minute, BP 80/40, 02Sat 96%.

No Previous Medical History.

Presenting History given as trauma call from Ambulance Control
7-year-old boy hit by a car which did not stop whilst he was crossing a road with older brother on a zebra crossing. Reported as unconscious briefly at scene by bystander then awake, agitated and confused. Ambulance arrived at scene within 7 minutes, child agitated and confused lying in the road. Airway patent, breathing, GCS 13. On route to hospital has become less responsive and drowsy on face mask oxygen. No Previous Medical History known.

Estimated Time of Arrival (ETA): 5 minutes

#### MIST hand over from Paramedics (observations from ambulance 5 minutes ago) to team

Mechanism: Struck by car as crossing a zebra crossing, speed and details unknown.

Injury: Abrasion to right arm and leg, small cut and abrasion to right side of head.

Signs: Pulse rate 140/min, respiratory rate 18/min, BP80/40, capillary refill 3 seconds. AVPU=V, SpO2 96 on 15 litres/minute of oxygen.

Treatment: Oxygen via facemask, C spine boarded from scene, IV access 22G left saphenous vein, IV Saline 10mls/kg bolus running.

*Observations on transfer to resuscitation bed*

Airway patent

SpO_2_ 96 %

Spontaneous ventilation, respiratory rate 16/min

HR 120 /min, BP 80 /40

CRT 3 seconds

GCS 8, P on AVPU

Pupils R=9 unreactive L=3 reactive

Temp 35C

*Available blood results:*

Venous blood gas 1 (on cannulation)

pH 7.27, pC02 5.9, P02 12.3, BE-4, Bic 22, Lactate 1

Blood glucose 6.2

Hb 11.8

FBC, UEs, X match sent – no results available

**10. Patient Demographics**

Age: 7Y
Weight: 35 kg
Gender: Male

**11. Equipment Requirement**

Gaumard Hal Simulator is fully dressed. A 22 G cannula is sited in left tibia, with tubing to allow injection into (tubing goes to receptacle under bed). Simulator has oxygen mask with tubing in place. Simulator is on a spinal board with head straps. Simulator has pulse oximetry monitor, ECG lead and BP cuff in place. Gaumard monitor to be used.

Paperwork to be given over if investigation done: a normal CXR image (laminated), a blood gas result. Parent role is played by confederate.

**12. Standardised Timeline**

| On arrival & handover | Parameters set at baseline | SpO_2_ 96 %, Spontaneous ventilation  Airway patent  Resp Rate 16/min  HR 120 /min, BP 100/50  CRT 3 seconds  P on AVPU  GCS 8 E=4, V1 (previously talking) M3  Pupils R=9 unreactive L=3 reactive  Temp 35C |
| --- | --- | --- |
| At 10 minutes | Stage 2 | SpO_2_ 98 %, Spontaneous ventilation  Resp Rate 10/min  HR 90 /min, BP 120/60  CRT 2 seconds  GCS 7 E3 V1 M3  Pupils R=9 unreactive L=3 reactive  Temp 36C |
| At 15 minutes  (If not intubated  continue with raised intracranial pressure signs) | Stage 3 | SpO_2_ 95 %, Spontaneous ventilation  Resp Rate 8/min  HR 70 /min, BP 140/70  CRT 2 seconds  GCS 3 E1 V1 M1  Pupils R=9 unreactive L=3 reactive  Temp 36C |
| Intubated | Stage 4 | SpO_2_ 97 %  Resp Rate Intubated and Ventilated  ETCO2 6.4  HR 80 /min, BP 120 /60  CRT 2 seconds  Pupils R=9 unreactive L=3 reactive  Temp 36.1 |
| Not intubated | Stage 5 | SpO_2_ 95 %, Spontaneous ventilation  Resp Rate 8  HR 65/min, BP 150/80  CRT 2 seconds  GCS 3 E1 V1 M1  Pupils R=9 unreactive L=3 reactive  Temp 36C |
| At 20 minutes  Not intubated No neuroprotective measures | Stage 6 | SpO_2_ 95 %, Spontaneous ventilation  Resp Rate 8/min  HR 60 /min, BP 170/80  CRT 2 seconds  GCS 3  Pupils R=9 unreactive L=3 not reacting |
| At 20 minutes  Intubated Neuroprotective measures | Stage 7 | SpO_2_ 97 %  Resp Rate Intubated and Ventilated  ETCO2 6.4  HR 80 /min, BP 120 /60  CRT 2 seconds  Pupils R=7 unreactive L=3 reactive  Temp 36.1 |
| At 20 minutes  Intubated No neuroprotective measures | Stage 8 | SpO_2_ 97 %  Resp Rate Intubated and Ventilated  ETCO2 6.4  HR 70 /min, BP 140 /70  CRT 2 seconds  Pupils R=9 unreactive L=3 reactive  Temp 36.1 |

| Continue as above after 20 minutes to end point | Stage 9 | Parameters unchanged as per each stage |
| --- | --- | --- |
| End point  At 30 minutes | Stage 10 | Aim is 30 minutes.  Sooner if:   1. Management of airway/ RSI with cervical spine immobilisation 2. Management of raised ICP and neuroprotection strategies 3. Performance of secondary survey 4. Prompt organisation of transfer to MTC |

**13. Expected Progression of Simulation**

| Trauma Call: By confederate to Emergency Department Red (Trauma) Phone | |
| --- | --- |
| Ambulance control handover of incoming child | Trauma Team called  Team roles identified  Team leader identified  Crashcall.net drug calculation sheet  Calculations recorded on board |
| Baseline (5 minutes after Trauma Call ends): Paramedic (confederate) arrives in ED bay at bedside (Time=0 ) | |
| Paramedic (confederate) 5 minutes after Trauma Call:  Arrive in ED at bedside (Time=0 ) Rapid MIST hand-over (<30 secs) | |
| Phase 1: Initial Assessment & Management | |
| Take MIST handover 30 secs  Trauma Team Leader establishes immediate status of child | |
| Primary Survey initiated by allocated team members | |
| Primary Survey feedback to Trauma Team Leader by Team | |
| Trauma Team Leader problem declares “Time-Critical Head Injury”; no other obvious injuries | |
| Team prepares to intubate & neuro-protect patient  Considers contacting Paediatric Major Trauma Centres and transfer team | |
| Trauma Team Leader contacts the Major Trauma Centre (MTC) to notify & organise CT scan at MTC | |
| Management of parent (confederate)  Leader acknowledges & briefs parents  Member of nursing staff allocated  Offer given to sit adjacent | |
| Phase 2: Deterioration | |
| Transition over 5 mins:  Patient’s BP rises, HR falls and RR falls. | TTL & Team recognises signs of raised intracranial pressure and problem is declared. |
|  | Prepares to intubate child activating team to gather required equipment |
|  | Requests for mannitol/3% hypertonic saline to be given or discussion with neurosurgeon or regional transport team for advice |
| Phase 3: Intubated, ventilated & neuroprotection | |
|  | Appropriate sedation and paralysis |
|  | Optimise ventilation |
|  | Head elevated to 30’, Systolic pressure approx.= 100, no pressure on neck veins, mannitol/3% hypertonic saline given. |
| Phase 4: Transfer to MTC | |
|  | TTL directs secondary survey  Organise time critical transfer to MTC  Category 1 Ambulance & appropriate transfer team |

**14. Key Debrief Points**

1. Recognition & management of closed head injury

2. Recognition & management of raised intracranial pressure

3. Team working

4. Handovers

**15. Disclaimer**
The cited information regarding the patient, in terms of the scenario and patient demographics is not an actual patient. Any resemblance to real person living or deceased will be coincidence.

**16. Reporting guidelines for health care simulation research**

| **Elements*** | **Sub-elements†** | **Descriptor** |
| --- | --- | --- |
| Participant orientation | Orientation to the simulator | On arrival trauma team members were informed that a simulator was replacing a real child and to perform as they would naturally do so. If there were any fidelity questions, they were instructed to ask a confederate (operating the simulator). |
|  | Orientation to the  environment | The environment was the participants’ daily workplace (an emergency bay in the emergency department). |
| Simulator type**16** | Simulator make and model | Gaumard Hal (5 year-old Hal) |
|  | Simulator  functionality | Fully functional with pupillary constriction/dilation capacity. No modifications. |
| Simulation environment**16** | Location | Emergency bay of each hospital. |
|  | Equipment | All normal trauma resuscitation equipment was used. |
|  | External stimuli | All background noises as per a normal trauma resuscitation were evident. |
| Simulation event/scenario**16** | Event description | The scenario, progression and triggers are described above. |
|  | Learning objectives | The learning objectives are described in the above scenario design. |
|  | Group vs individual  practice | Trauma teams. |
|  | Use of adjuncts | Simulator was dressed to resemble a 7-year-old child that had become injured. On arrival the child was spinal boarded with collar, fully monitored, with oxygen mask and a working cannula (for head injury scenario). |
|  | Facilitator/operator  characteristics | Three facilitators/operators were present at each simulation, two experienced (each 15-20 years) simulation and paediatric trauma practitioners (first two authors) and a simulation research coordinator. Post-simulation debriefs were all performed by the first author. |
|  | Pilot testing | Twenty pilot simulations were conducted as described in the methodology. |
|  | Actors/confederates/  standardised/  simulated patients**16** | For the head injury scenario, one of the first two authors played the role of a paramedic who entered ED dressed as a paramedic with the simulator on a paramedic trolley as described above and provided the MIST handover. The computer was operated by the research coordinator or an initial confederate who had left the scenario. |
| Instructional design (for educational interventions)**19** or exposure (for simulation as investigative methodology)**16** | Duration | Thirty minutes was allocated for each simulation and debrief. The time-critical head injury scenario ended when the simulator was appropriately managed (intubated and ventilated) and being readied to leave ED for definitive care/CT if at the Major Trauma Centre. Twenty minutes was provided to reach this end point or not.  Immediately post the simulations, participants were given a team performance checklist to record teamwork and their comments (as described in the methodology). Then a debrief was delivered by the first author. |
|  | Timing | Prior to the simulations a site visit occurred at each venue. All simulations were planned for a specific date and time (0900) at each of the trauma unit hospitals in the study. The pilot simulations at the Major Trauma Centre were also between 0900 to 1200. The standardised arrangements to veto a simulation remained at each site. |
|  | Frequency/repetitions | There was no repetition of any simulations. |
|  | Clinical variation | The simulations were standardised as described above. |
|  | Standards/assessment | Participants were trauma team members allocated to each trauma team as part of their normal daily working. |
|  | Adaptability of intervention | The intervention was standardised. |
|  | Range of difficulty | The scenarios were standardised. |
|  | Non-simulation interventions and  adjuncts | Not used as part of this study. |
|  | Integration | There was no integration into a curriculum |
| Feedback and/or debriefing**11** | Source | The simulator provided all clinical pathophysiological feedback, except for capillary refill. This was provided by a confederate. |
|  | Duration | As detailed above, thirty minutes was allocated for each simulation and debrief. |
|  | Facilitator presence  Facilitator  characteristics | Facilitator presence and experience is described above. |
|  | Content | The content was designed to develop an assessment and feedback tool focusing upon the interplay between individuals, the trauma team and the hospital system as described in the manuscript. |
|  | Structure/method | Debriefing focused upon two key clinical and non-technical skill factors per scenario. The debrief occurred after both scenarios and was situated in the Emergency Department. The first author facilitated each debrief. |
|  | Timing | As above. The debrief duration was approximately ten minutes per scenario. |
|  | Video | No video. |
|  | Scripting | No scripting of debrief was employed. |
