# Supplementary material for: A novel approach to explore Safety-I and Safety-II perspectives in in situ simulations—the structured what if functional resonance analysis methodology
Source: Adv Simul (Lond). 2021 Jun 5;6:21. doi: 10.1186/s41077-021-00166-0 (PMC8178899; doi:10.1186/s41077-021-00166-0)
Supplement: Supplementary file 2 — Additional file 2. The construction of FRAM models of work-as-imagined (WAI) and work-as-done (WAD) and the SWI-FRAM approach. [file 41077_2021_166_MOESM2_ESM.docx]

# SUPPLEMENTARY FILE 2. THE CONSTRUCTION OF FRAM MODELS OF WORK-AS-IMAGINED (WAI) AND WORK-AS-DONE (WAD) & THE SWI-FRAM APPROACH

The reader is directed to a complete online resource for the construction of FRAM models (24). Below is intended as a short introduction to the ideas and steps in FRAM modelling. It basically consists of a sequence of logical steps.

## 1. What are we modelling?

Here we build a data and information base about the process involved. This can consist of a mixture of desk work and interviews with the actual people who know / operate these systems. For example.

- What tasks are involved?
- What functions are needed to perform those tasks?
- Are there critical needs for the process to complete successfully?
- Do we have the timings, sequencing, resources, preconditions, mandatory controls, external constraints, operational limitations, etc., needed to enable the system to perform as designed?

## 2. How do these functions combine to define the system?

The aim here is to build a FRAM model of the system involved in the process/activity. So, the functions identified, are set out in an interacting, interdependent picture, as a “cloud” visualisation, normally achieved using the software tool, the FRAM Model Visualiser, (FMV)(25).

- For each function, we need to define the critical “FRAM aspects”, or execution conditions (Figure 1 above), needed to allow the function to operate and fulfil its task.
- These “Aspects” will thus consist of an **Input (s**) needed to trigger the function, plus any other requirements, such as **Preconditions**, **Resources**, **Control** states or signals and any T**imin**g constraints.
- These interacting links between the functions can only arise as **Outputs** of other functions and as such, they will build up as the analyst steps through all the functions in the system.
- Functions with O**utputs** only, are, by definition, **Background** functions, which set the boundaries of the system. All the rest are therefore **Foreground** functions.
- In a sequence of tasks in a particular instantiation of the model, (a specific instance or “snapshot” of the system’s operations), those functions which have to deliver before others can begin, are called **Upstream** functions.
- Then subsequent functions are obviously **Downstream** functions (Figure 3).

Figure 3. Up and Downstream functions in a FRAM Model


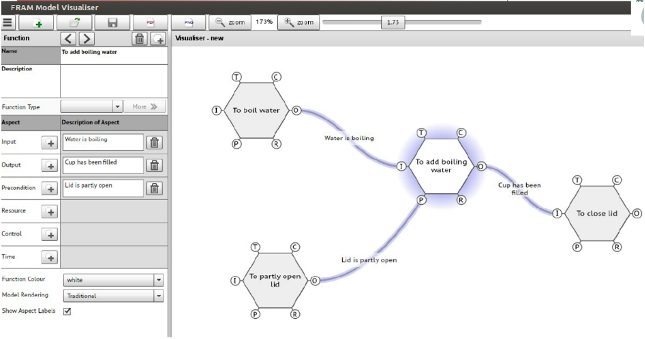


In this FRAM model, “to boil water” is an upstream function and “to close lid” is a downstream function.

- In any particular **instantiation** of the model outputs from downstream functions cannot initiate upstream functions if they themselves have not been initiated or until they themselves are initiated.
- Similarly, **closed loops** are not allowed, where a function’s **outputs** are linked back as aspects of the same function.
- In contrast, aspects not linked to specific **outputs** of other functions are known as “**Orphans**”, which need removing before a model can be validated.

## 3. Testing the System Model’s validity

Normally this “As Imagined” system model is checked in two ways:

- **Peer Review** – Most FRAM analysts will, at this stage, look for confirmation of the model’s accuracy and check the visualisation as a run through session with the teams involved, Figure 4.

Figure 4. An example FRAM model


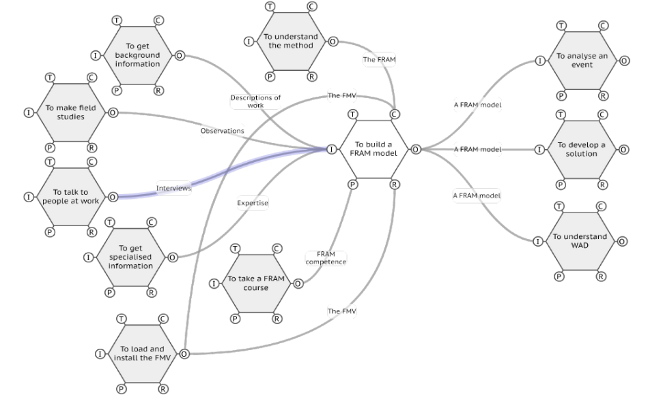


This is a relatively simple FRAM model where all functions can be easily checked in a peer review process.

- **Formal Validation** – Having agreed that the system model now accurately reflects the process “As Done”, the FRAM model can now be checked and adjusted for consistency and completeness, using the FRAM Model Interpretation (FMI) (19).

## 4. Gaining insights from observed and expected variabilities in these systemic interactions?

There are two basic ways a FRAM modelled system is used to achieve these insights:

- **A. From observations on actual or simulated work situations**, where the FRAM visualisation now allows a recording and analysis of the effects of actual variabilities in the status of the necessary links, in real environments.
- In working through the effects of these variabilities, the analyst and the work teams can then elicit and identify elements and situations where there are opportunities to correct and compensate for problems and enhance/ enable more successful and resilient operations.
- **B. By a formal process**, where systematically, starting with the entry function, the question is posed, what if the output of this function is normally, in terms of timing, on time, what happens to the system if it’s too early, too late or not at all,
- Similarly, in terms of precision (scale, quantity, etc,) what happens if the output of that function is instead of being precise is variable within acceptable limits, or imprecise. This is shown in Figure 5.

Figure 5. A SWI-FRAM example.


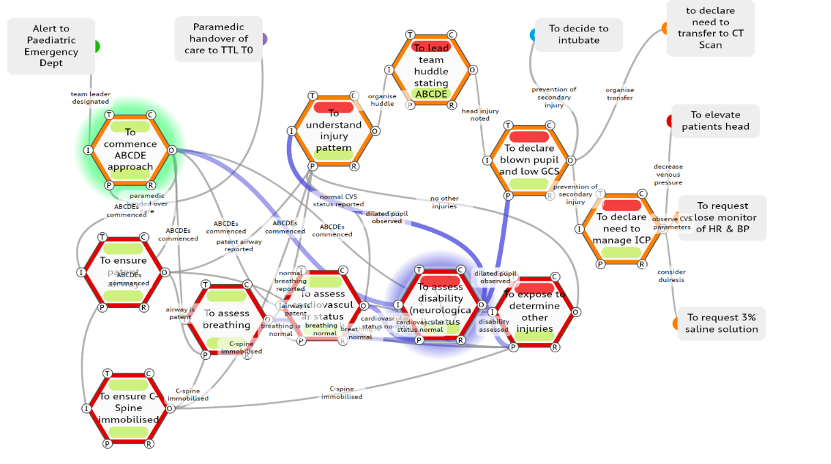


In this example the ABCDE approach is underway successfully (functions are completed on time & with precision, green top and bottom) however if the pupils were not examined, the functions shown in red could not be completed successfully.

- Again, the FMI software tool allows the analyst to highlight and identify the consequential and often unexpected emergent downstream effects of these variabilities on other functions (the resonances implied by the FRAM acronym), and hence the system’s expected performance.

## 5. Applying the insights gained

As we now have a validated and consensual “model” of the system actually employed in practice, the analyst, and / or the team, can start to look more closely at the system. It thus allows them to incorporate the lessons learned from what affects the system, both negatively (SAFETY I) and positively (SAFETY II). This is the opportunity to modify the system, not only to eliminate the constraints and shortcomings, but also to incorporate those adaptations, that the work teams have adopted, as ways of working around, optimising operability and thus improving productivity and safety.
