# Supplementary figures and images for: A novel approach to explore Safety-I and Safety-II perspectives in in situ simulations—the structured what if functional resonance analysis methodology
Source: Adv Simul (Lond). 2021 Jun 5;6:21. doi: 10.1186/s41077-021-00166-0 (PMC8178899; doi:10.1186/s41077-021-00166-0)

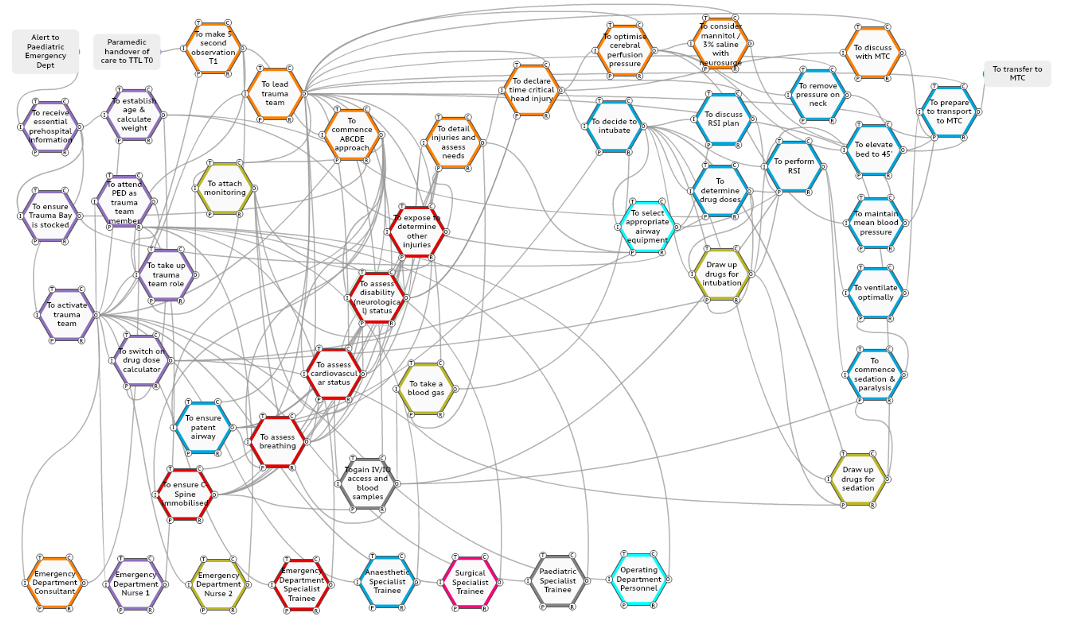

Supplement: Supplementary file 3 — Additional file 3. WAI. [file 41077_2021_166_MOESM3_ESM.png]
